# Unsupervised seamless UAV image stitching via dense prediction

**DOI:** 10.1371/journal.pone.0348616

**Published:** 2026-05-14

**Authors:** Jun Chen, Honghua Tang, Dongmei Yan, Shuo Chen, Jie Wang, Jianhui Li

**Affiliations:** 1 Aerospace Information Research Institute, Chinese Academy of Sciences, Beijing, China; 2 School of Computer Science and Technology, University of Chinese Academy of Sciences, Beijing, China; 3 Computer Network Information Center, Chinese Academy of Sciences, Beijing, China; 4 School of Electronics and Communication Engineering, Guangzhou University, Guangzhou, China; 5 School of Frontier Sciences, Nanjing University, Suzhou Campus, Suzhou, China; Leibniz University Hannover, GERMANY

## Abstract

Image stitching is a critical preprocessing step in unmanned aerial vehicle (UAV)-based remote sensing, however, it remains challenging for conventional methods due to large parallax, high resolution, and scene complexity, which often lead to visible seams or high computational overhead. To address these challenges, an unsupervised, mask-based image stitching approach is proposed in this study. The proposed approach reframes UAV image stitching as a 2D dense prediction and assigns the optimal pixel from source images to every pixel in the overlap. The proposed approach comprises two key components, i.e., Siamese-Residual Mask Network (SRMN) and Accelerated Inference via Scale Decoupling (AISD). The SRMN component leverages a Siamese architecture to extract multi-level features and compute residual discrepancies, guided by loss constraints of spatial consistency, smoothness, and quality-aware to generate seamless binary masks. By exploiting the strong entropy stability of binary masks across scales, the AISD component predicts masks at a low resolution. This allows for the accurate restoration of full-resolution masks for blending without requiring network retraining or structural changes. By decoupling mask prediction from high resolution processing, the proposed approach avoids expensive nonlinear operations while preserving fine details. Experiments on four UAV image datasets show that the proposed approach outperforms state-of-the-art methods. The proposed approach improves PSNR by up to 28.58% and SSIM by up to 56.00%, while reducing processing time by up to 88.9% compared to state-of-the-art methods. These results validate the proposed approach as a robust solution for large-scale UAV image stitching, successfully balancing computational efficiency with visual fidelity.

## Introduction

UAV remote sensing has become an important tool for Earth observation due to its high maneuverability, low cost, and ability to acquire centimeter-level ultra-high-resolution imagery [1,2]. Its successful applications in agricultural monitoring [3,4], infrastructure inspection [5–7] and disaster response [8–11] highlight its value in rapidly providing high-quality ground information. However, limited by flight altitude and camera field of view, a single UAV image cannot cover the entire target area. Therefore, image stitching to generate panoramic views has become a critical step in UAV remote sensing data processing.

A key challenge in this process is the significant parallax between UAV images, arising from low flight altitudes and scene depth variations, which often leads to stitching artifacts and geometric misalignment [12–15]. Additionally, positional shifts of dynamic subjects (such as moving vehicles and pedestrians) across different images may further disrupt pixel correspondence. These factors make it particularly difficult to achieve geometric robustness and dynamic scene adaptability in image stitching.

In response to these challenges, existing parallax image stitching methods can be broadly divided into two categories. The first type enhances the degrees of freedom of geometric transformation models (e.g., local alignment) to improve registration accuracy [16–19]. These methods perform well under small parallax conditions but struggle with large-baseline images. To preserve image structure in wide-baseline scenarios, LPC [20] proposed a seam-driven matching strategy based on line-point consistency, which significantly improves stitching quality at the cost of increased computational time. The second type of methods bypasses the challenge of precise alignment and instead focuses on optimizing the optimal seam path to suppress ghosting and visible stitching artifacts [21–24]. These approaches construct energy functions based on pixel-level differences (e.g., color, gradient) and employ dynamic programming or graph-cut algorithms to search for the seam with minimal visual discrepancy. Since they do not rely on perfect alignment, they are particularly suitable for UAV image stitching with significant parallax and complex 3D structures [25–27]. For large-scale orthophoto mosaicking tasks with hundreds of images, similar seamline optimization principles have been extended to global multi-image scenarios [28,29].

However, such seam optimization methods inherently simplify the two-dimensional visual consistency problem into a one-dimensional continuous path search, leading to intrinsic limitations: their solution space is constrained to a single continuous curve, making them prone to local optima; meanwhile, the topological connectivity of the seam path makes it difficult to avoid dynamic objects or regions with depth discontinuities, often resulting in ghosting artifacts or structural breaks. The fundamental reason lies in the fact that image stitching is essentially a two-dimensional, pixel-level decision process—each pixel in the overlapping region should be independently assigned to its source image. Traditional methods reduce this problem to a one-dimensional path search, neglecting the local independence and spatial non-continuity of pixel correspondences under multiple viewpoints, thereby limiting the expressive capacity of the solution space and hindering high-quality stitching in complex scenes.

Traditional methods have inherent limitations, particularly their inability to perform independent pixel-wise decisions and their lack of semantic understanding. Given these limitations, deep learning offers a promising alternative. Unlike handcrafted features and predefined energy functions, deep neural networks can learn robust feature representations directly from data and implicitly model scene semantics, enabling them to differentiate between static structures (e.g., buildings, roads) and dynamic objects (e.g., moving vehicles). This capability is crucial for handling the challenges of large parallax and dynamic scenes, because it allows for more flexible, content-aware stitching strategies that go beyond the constraints of a single continuous seam.

Recent studies have attempted to introduce deep learning into image stitching, which can be categorized into three types. The first category focuses on improving the robustness of image alignment. Some works [30–33] design end-to-end networks to directly regress accurate homography matrices, while others [34,35] aim to enhance the robustness of image features. The second category emphasizes improving fusion quality. Some approaches leverage deep learning to extract high-level semantic information, enabling the seam to avoid semantically meaningful regions such as vehicles or pedestrians [36,37]. Others focus on detecting geometric structures, using deep learning to identify object contours and prominent edges, thereby ensuring that the seam avoids salient objects and strong edges, minimizing visual artifacts and maintaining structural integrity [38]. Although conceptually advantageous, these methods heavily rely on high-quality semantic annotations, facing multiple practical challenges: high annotation costs, class imbalance, and limited generalization capability in complex open-world scenarios (e.g., UAV remote sensing) [39]. The third category aims at end-to-end image stitching. Supervised methods [40,41] are trained using synthetic data or results from traditional methods as ground truth, but suffer from poor generalization and fundamental difficulties in acquiring reliable ground truth. Unsupervised methods [42–44] do not require labeled data, yet pose greater challenges in training and optimization. Moreover, regardless of the type of deep learning model, directly processing the ultra-high-resolution images commonly captured by UAVs leads to a dramatic increase in GPU memory consumption and computational load, severely limiting their practicality.

Despite these diverse and promising attempts, most existing deep learning-based stitching methods still operate within conceptual frameworks inherited from traditional techniques. Specifically, methods in the first category remain focused on improving alignment accuracy, a goal that is fundamentally limited by the inherent ambiguity of pixel correspondences under large parallax. The second category, while introducing semantic or geometric guidance, still adheres to the one-dimensional seam paradigm, thereby inheriting its fundamental constraint: the solution space is restricted to a single continuous path. The third category aims for end-to-end stitching, yet faces persistent challenges in obtaining grouth truth(for supervised methods) or training stability (for unsupervised methods), and all three categories struggle with the computational demands of ultra-high-resolution UAV imagery. These observations suggest that advancing the field may require not just incremental improvements within existing frameworks, but a fundamental rethinking of the stitching problem itself.

In summary, although existing methods have made certain progress in handling parallax and dynamic scenes, significant challenges remain. In traditional methods, local alignment often requires introducing strong geometric constraints to cope with large parallax, leading to a dramatic increase in computational burden. While optimal seam methods reduce the need for precise alignment, their dimensional reduction of the two-dimensional stitching problem into a one-dimensional path search severely restricts the solution space, limiting flexibility in handling spatially discontinuous regions. Moreover, their reliance on hand-crafted energy functions lacks the capability to deeply model semantic structures and visual consistency. In recent years, deep learning has introduced new technical perspectives for image stitching. However, most of these methods are still constrained by the trade-off among annotation cost, model generalization, and computational efficiency, making them difficult to directly apply to UAV image stitching tasks involving high resolution and large parallax.

This paper proposes an unsupervised dense prediction-based stitching method that transforms the traditional one-dimensional seam optimization problem into a more fundamental two-dimensional pixel-level classification task. Based on this, we construct an end-to-end deep learning framework that directly assigns the optimal source image to each pixel within the overlapping region, thereby generating physically plausible and visually coherent stitching masks. This approach not only fundamentally aligns with the two-dimensional decision-making nature of image stitching, but also eliminates reliance on manually annotated data, enhancing applicability in real-world complex scenarios, and providing a novel solution for efficient and robust processing of UAV images with large parallax.

In implementation, we adopt an encoder-decoder architecture to overcome the limitations of traditional single-scale difference metrics (e.g., color, gradient) in semantic perception and feature hierarchy. Specifically, we employ dual encoders to extract multi-scale features from the reference and target images respectively, effectively capturing deep semantic information and fine-grained texture details. Cross-scale feature fusion is achieved through skip connections, thereby enhancing the model’s ability to perceive visual differences in complex scenes. During training, physical priors such as local consistency and boundary smoothness are encoded into differentiable loss functions to constrain potential fragmentation and discontinuities caused by independent pixel-wise predictions [45,46], further improving the visual coherence and structural rationality of the stitching results.

The main contributions of this paper are threefold.

1) We propose a new formulation that models image stitching as a dense prediction task. We reformulate stitching from a one-dimensional seam search to a pixel-level classification task, where each pixel in the overlapping region is independently assigned to its source image. This formulation fundamentally overcomes the limited solution space and topological rigidity inherent in traditional optimal seam methods, enabling more flexible handling of depth discontinuities and dynamic objects.
2) We present an unsupervised mask learning paradigm with a dedicated Siamese-Residual Mask Network (SRMN). An end-to-end neural network with three physics-informed loss functions is designed to learn stitching masks directly from data without manual annotations. This eliminates reliance on hand-crafted features and energy functions in traditional methods, as well as the annotation dependency in supervised deep learning approaches.
3) We achieve accelerated inference through a newly developed scale decoupling strategy. Based on the observation that binary masks preserve geometric structures under resolution scaling, we propose a method to accelerate network inference by allocating computationally expensive nonlinear mask prediction and efficient linear blending to different resolution spaces. This preserves fine-grained details while avoiding high-cost full-resolution nonlinear computation with no need for network retraining or architectural changes.

The remainder of this paper is organized as follows: First, the datasets used in this paper and the proposed method are introduced. Next, the experimental results are presented. Then, an in-depth discussion of the proposed method is conducted. Finally, the paper is concluded.

## Methods

### Overview of the proposed method

**Fig 1:**
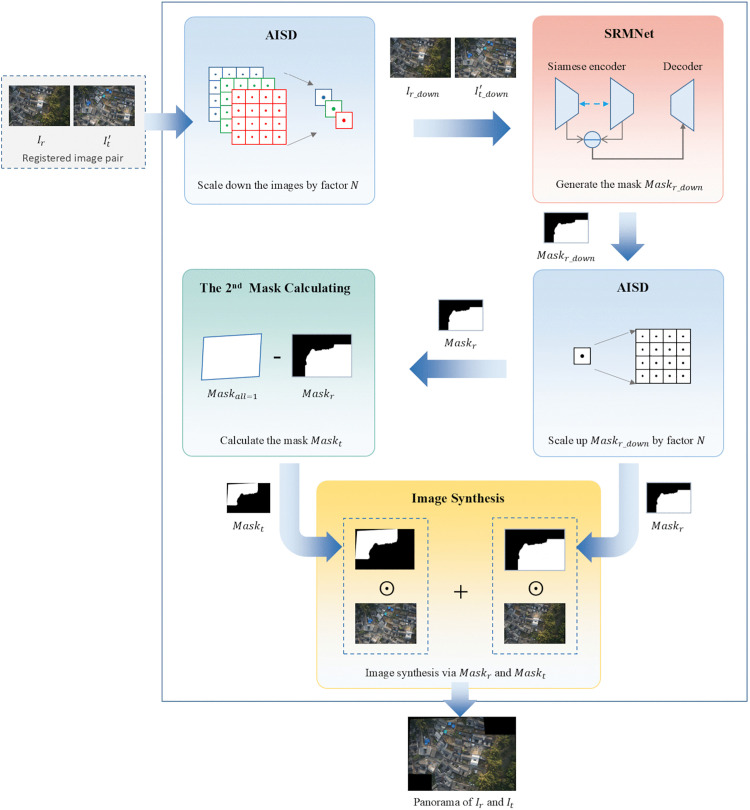
Schematic diagram of unsupervised learning image stitching method driven by multi-level feature differences.

This study formulates the image stitching problem as a pixel-level dense prediction task, generating high-resolution stitching masks through end-to-end learning. The proposed method incorporates three key designs: First, we design a Siamese-residual mask network(SRMNet), which is an unsupervised mask generation network based on a Siamese encoder-residual decoder architecture. It employs a weight-shared dual-branch encoder to extract multi-scale features across images, and the feature difference awareness is then achieved through hierarchical residual fusion for the outputs of the image stitching mask. Second, we propose a composite loss function. This loss is composed of a spatial extent term, a smoothness term, and a quality-aware term. By using this loss, we transform many physical priors, such as image spatial constraints, cross-boundary continuity, and registration quality, into differentiable optimization objectives, guiding the network to generate spatially coherent and visually plausible stitching masks. Third, we introduce the Accelerated Inference via Scale Decoupling (AISD) strategy to address the computational burden and memory pressure caused by high-resolution drone imagery. In this strategy, computationally intensive mask prediction is performed in low-resolution space, followed by upscaling to restore the original resolution. In high-resolution space, mask-guided weighted fusion and other linear operations are implemented. The overall framework of the proposed method is illustrated in Fig 1. Following the AISD strategy, the registered input images $I_{r}$ and $I_{t}$ are first downsampled to low-resolution counterparts $I_{r,down}$ and $I_{t,down}$, and they are then fed into SRMNet to predict a low-resolution stitching mask $Mask_{r,down}$. This low-resolution mask is subsequently upsampled to the original resolution, yielding the high-resolution mask $Mask_{r}$. The corresponding mask for $I_{t}$ is computed by $Mask_{t}=1-Mask_{r}$. Finally, the stitched result is produced by fusing $I_{r}$ and $I_{t}$ using $Mask_{r}$ and $Mask_{t}$, resulting in a seamless composite, given by


$$\begin{array}{c} I_{M}=I_{r}⊙Mask_{r}+I_{t}⊙Mask_{t}\;, \end{array}$$ (1)

where ⊙ represents Hadamard product, which is also known as element-wise product.

In the following three parts, we first provide the details of the SRMNet, then introduce the composite loss function design, and finally present the AISD strategy.

### Siamese-Residual Mask Network

**Fig 2:**
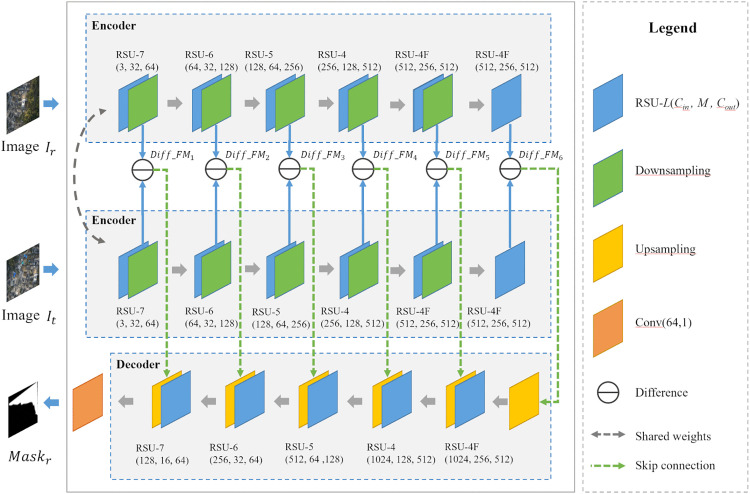
Framework of the Siamese-residual mask network.

We propose an unsupervised learning-based network for image stitching mask generation, utilizing a Siamese encoder-residual decoder architecture to enable hierarchical feature difference perception. By incorporating composite constraints, the proposed method aims to overcome the one-dimensional path limitation inherent in traditional seam-line search methods. As illustrated in Fig 2, the core structure comprises a Siamese encoder for cross-image multi-scale residual modeling and a residual-driven decoder. In this structure, precise mask generation is accomplished through feature space alignment and cross-layer information fusion.

For the encoder, this study implements a Siamese framework with weight-shared dual branches to extract multi-scale features from input image pairs separately. Hierarchical feature residuals are computed across branches to capture scene discrepancies. The shared-weight Siamese architecture enforces both input images to embed into a common feature space, ensuring that the computed feature residuals reflect only genuine scene content differences while eliminating bias caused by network asymmetry. Each branch of the encoder comprises N cascaded Residual U-blocks (RSU) [47] integrated with downsampling units. The RSU-*L*($C_{in}$, *M*, $C_{out}$) module constitutes a depth-tunable U-shaped nested structure for multi-scale feature fusion.

**Fig 3:**
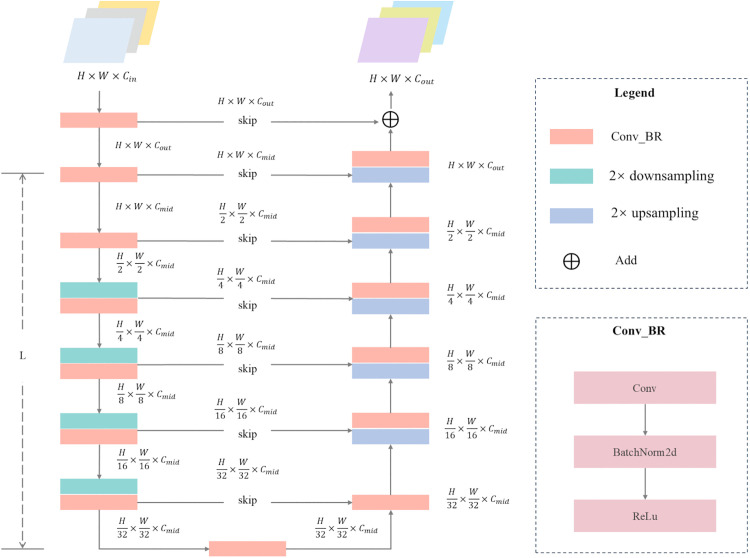
Schematic diagram of the RSU block structure.

As illustrated in Fig 3, the RSU module employs *L* levels of nested Conv_BR blocks, where each block includes convolution, batch normalization, and ReLU activation, to construct an symmetric contracting-expanding encoder-decoder architecture. The input feature is first progressively downsampled through multiple stages, gradually expanding the receptive field to capture global semantic information. Subsequently, a symmetric upsampling path restores spatial resolution, while features from corresponding encoder levels are concatenated across scales and fused to produce the output. This enables the integration of fine-grained local details with rich global context. The depth parameter *L* controls the feature extraction range. As the value of *L* increases, the nesting hierarchy deepens, and the number of pooling operations also increases synchronously. The input and output channel dimensions are defined by $C_{in}$ and $C_{out}$, respectively, while the intermediate channel number *M* regulates model capacity. These three parameters jointly balance computational efficiency and feature representation capability. This adaptive architecture preserves the original spatial resolution throughout the process and mitigates the detail degradation typically caused by single-step upsampling, thereby significantly enhancing edge and boundary preservation in the generated features.

The *N* layers of RSU modules in the encoder are designed with progressively reduced depth to enable multi-scale feature extraction from coarse to fine levels. The first stage employs an RSU-7 module, aiming to extract global base features through seven levels of nested downsampling and max-pooling operations. Intermediate layers sequentially deploy RSU-6, RSU-5, and RSU-4 modules, progressively reducing the depth of hierarchical recursion to focus on finer local structures. In the deeper layers, an enhanced RSU-4F module is introduced to replace conventional downsampling with dilated convolutions at dilation rates d∈{2,4,8}. This enables exponential expansion of the receptive field without altering the spatial resolution of feature maps. This design enhances contextual awareness through multi-scale sampling via dilated kernels, significantly enriching the semantic completeness and geometric consistency of deep features without significantly increasing the number of parameters. The resulting high-fidelity feature representations provide precise discrepancy cues for the subsequent decoder.

The decoder adopts a single-branch architecture, reconstructing features through a cross-level residual propagation mechanism. Each decoding stage consists of an RSU module configured with the same depth as its counterpart in the encoder, followed by an upsampling unit, ensuring architectural symmetry between the encoder and decoder paths. This study introduces a skip connection mechanism that directly injects feature residuals *Diff*-$FM_{i}$ defined as the difference between features extracted from the two encoder branches at level I into corresponding decoder layers. By fusing cross-level difference features that range from fine-grained details in shallow layers to high-level semantics in deep layers, this mechanism enhances multi-scale difference representation and effectively mitigates gradient vanishing. During training of the deep network, the hierarchical difference information is preserved and utilized more comprehensively, significantly improving the sensitivity of the model to complicated scene variations and providing rich multi-level cues for mask generation. To generate the final stitching mask, the multi-channel output of the decoder is processed by a follow-up compression block Conv(64,1): a convolution reduces the channel dimension to one, producing the feature map $FM_{s}$, which is then normalized through a Sigmoid function to obtain the soft mask with its range from 0 to 1.

The proposed network has two benefits for the UAV image stitching task. First, the encoder constructs a hierarchical multi-scale feature representation through a cascade of RSU modules with decreasing depth, enabling progressive difference modeling from global structure to local texture. Second, the weight-sharing strategy within the Siamese architecture maps the dual-input streams into a unified feature space, effectively suppressing representational bias and ensuring that feature differences arise solely from genuine content variations in the input images. This encoder-decoder collaborative framework for multi-level feature difference perception significantly enhances the adaptability of mask generation to complicated application scenarios.

### Loss functions

Inspired by the spatial continuity optimization used in semantic segmentation, we design a differentiable composite loss function, aiming to guide the generation of stitching masks. The loss function is introduced after accounting for the following three constraints. The first constraint considers the spatial extension, and it enforces that the mask boundary lies within the image overlapping region and voids in non-overlapping areas can be avoided through the spatial position regularization. The second one considers cross-image smoothness, and this minimizes the image discontinuities across the mask boundary, enhancing the visual continuity of the final result. The last one considers registration quality, and we achieve this goal by selecting regions with smaller image registration errors, ensuring high alignment accuracy in the mask transition zone. These constraints are described one by one in detail, and we then introduce the proposed composite loss.

#### Spatial extent term.

**Fig 4:**
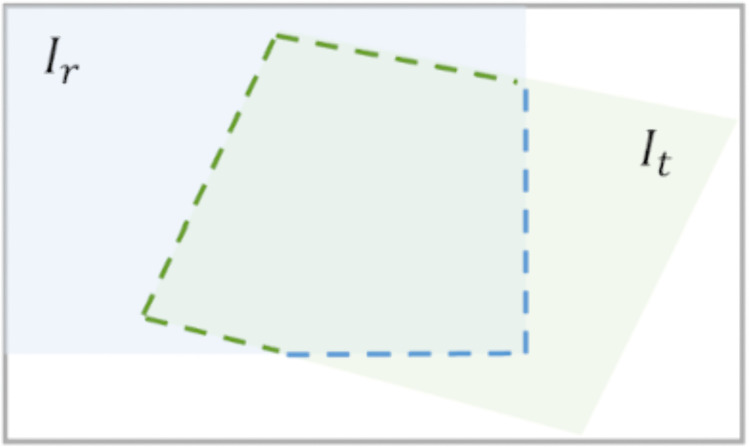
The two images to be stitched, $I_{r}$ and $I_{t}$, and their overlapping region $\Omega_{ov}$. The blue and green dashed lines represent the boundary segments of $I_{r}$ and $I_{t}$ within $\Omega_{ov}$, respectively.

The spatial distribution of the image stitching mask has the direct impacts on the integrity of the stitching result. If the mask exceeds the valid boundary of the corresponding image and invades the non-overlapping area of another image, it causes invalid pixels being erroneously merged, resulting in stitching holes. To address this issue, this paper proposes a spatial constraint for masks based on the boundaries of overlapping regions. This constraint utilizes the boundary information within the overlap region $\Omega_{ov}$ between two images to be stitched, $I_{r}$ and $I_{t}$, to limit the range of the mask. Specifically, it involves extracting the boundary segments of $I_{r}$ and $I_{t}$ within $\Omega_{ov}$, where they are illustrated in blue and green dashed lines in Fig 4, respectively, and then we binarize them to generate geometric constraint masks aimed at suppressing the out-of-bound behavior of the stitching mask, denoted as $Boundary_{r}$ and $Boundary_{t}$, respectively. If the stitching mask does not exceed the boundary of its corresponding image, the Hadamard product of the mask with its corresponding geometric constraint mask would yield zero.

**Fig 5:**
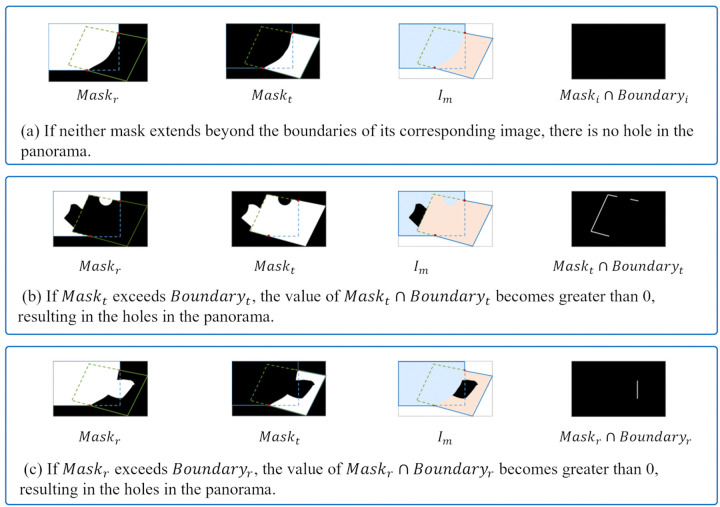
The impact of the relationship between the mask and the overlapping region on the stitching results.

Fig 5 shows the impact of mask boundary violations on the final stitching results, and two obvious phenomena can be observed. If the mask does not violate the boundary of its corresponding image, both $Mask_{t}⊙Boundary_{t}$ and $Mask_{r}⊙Boundary_{r}$ yield zero values, as shown in Fig 5(a), avoiding introducing artifacts to the final stitched result. If either mask extends beyond the boundary of its corresponding image, i.e., $Mask_{r}⊙Boundary_{r}\neq 0$ or $Mask_{t}⊙Boundary_{t}\neq 0$, it encroaches into the non-overlapping region of the other image, resulting in visible holes or missing content in the stitched output, as demonstrated in Fig 5(b) and 5(c).

The above analysis indicates that the spatial constraint can effectively avoid causing severe hole artifacts. Based on this constraint mechanism, this paper designs the loss function ${ℒ}_{extent}$ of spatial extent term, given by:


$$\begin{array}{c} \begin{array}{c} {ℒ}_{extent}=\sum_{i\in \{r,t\}}||Mask_{i}⊙Boundary_{i}|{|}_{1}, \end{array} \end{array}$$ (2)

where $||\cdot |{|}_{1}$ denotes the L1 norm, which is used to strengthen the zero-value constraint, and “⊙” represents the Hadamard product.

This loss function enforces the network to generate stitching masks that are strictly confined within the boundaries of the corresponding images through dual-boundary joint supervision. Moreover, it prevents pixel coverage imbalance caused by mask boundary overreach.

#### Smoothness term.

The smoothness term ${ℒ}_{smooth}$ serves to minimize the differences between the two images near the mask boundary. This paper employs the most common pixel value differences and incorporates gradient value differences to characterize the dissimilarity between images, which can be written as


$$\begin{array}{c} \begin{array}{c} {ℒ}_{smooth}=\sum_{i,j}\sum_{\delta \in \{(n,0),(0,n)\}}|Mask_{r}^{\left(i,j\right)}-Mask_{r}^{\left(i+\delta_{x},j+\delta_{y}\right)}|(D^{\left(i,j\right)}+D^{\left(i+\delta_{x},j+\delta_{y}\right)}), \end{array} \end{array}$$ (3)

where $\delta =(\delta_{x},\delta_{y})$ represents a two-dimensional integer displacement vector with n a positive integer, |·| denotes the absolute value operation, $Mask_{r}^{\left(i,j\right)}$ denotes the mask value at pixel coordinate (*i*,*j*), and *D*^(*i*,*j*)^ represents the composite difference metric between the two images. We further combine the intensity difference and gradient difference, then we have:


$$\begin{array}{c} D^{\left(i,j\right)}=||I_{r}^{\left(i,j\right)}-I_{t}^{\left(i,j\right)}|{|}^{2}+\alpha ||▽I_{r}^{\left(i,j\right)}-▽I_{t}^{\left(i,j\right)}|{|}^{2}. \end{array}$$ (4)

Here, $||I_{r}^{\left(i,j\right)}-I_{t}^{\left(i,j\right)}|{|}^{2}$ is the intensity difference at pixel (*i*,*j*), and $||▽I_{r}^{\left(i,j\right)}-▽I_{t}^{\left(i,j\right)}|{|}^{2}$ is the gradient difference at pixel (*i*,*j*). α is a balancing coefficient.

The purpose of introducing the gradient difference is to guide the boundary to lie within regions of smaller image gradients even when the pixel intensity difference is small. Compared with smooth regions, image alignment errors are more perceptually sensitive in regions with large gradients, and local structures also change drastically. Therefore, placing the stitching boundary in these areas may lead to noticeable visual artifacts.

When $Mask_{r}^{\left(i,j\right)}-Mask_{r}^{\left(i+\delta_{x},j+\delta_{y}\right)}=0$, this indicates that pixels (*i*,*j*) and $\left(i+\delta_{x},j+\delta_{y}\right)$ originate from the same image, and the loss ${ℒ}_{smooth}$ is zero. Otherwise, it means that they come from different images, and a penalty must be applied to the boundary between them. Summing the loss values over all pixels within the overlapping region yields the overall smoothness loss term.

#### Quality term.

Existing seam optimization algorithms mainly focus on intensity and texture differences between images, while paying less attention to the influence of registration errors. Such methods can improve visual continuity, but neglecting the impact of registration errors on geometric consistency may lead to local misalignments or stitching artifacts. As a matter of fact, registration errors have a significant effect on seam distribution [48]. For this consideration, this study introduces a geometric registration accuracy constraint to guide the mask away from regions with prominent edge structures and regions where registration errors exceed a predefined threshold, thereby balancing visual consistency with the geometric accuracy and robustness of the stitched result.

**Fig 6:**
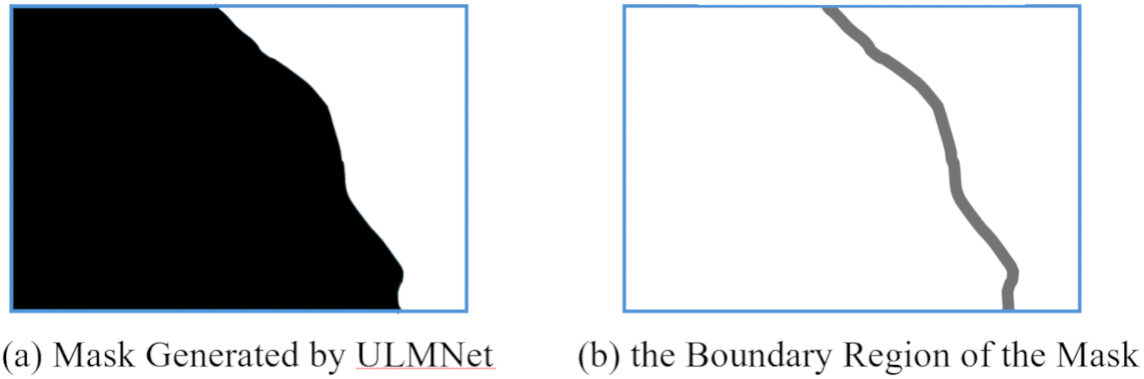
The mask generated by SRMNet and its boundary region.

Accordingly, this paper proposes a registration error-aware loss function, namely the quality term ${ℒ}_{quality}$, aimed at improving the image stitching quality assessment score within the mask boundary region. We adopt structural similarity (SSIM) [49] as the evaluation metric to design ${ℒ}_{quality}$, which is given by:


$$\begin{array}{c} \begin{array}{c} {ℒ}_{quality}=(1-SSIM(R_{b}))/2, \end{array} \end{array}$$ (5)

where $R_{b}$ is the mask boundary neighborhood. The image within $R_{b}$ is used to calculate the quality assessment score. Fig 6(a) shows the mask generated by SRMNet, and Fig 6(b) depicts its boundary region $R_{b}$. The SSIM score ranges from −1–1, while ${ℒ}_{quality}$ only ranges from 0 to 1, and a lower value indicates higher stitching quality. One can see that this loss function quantifies the structural fidelity in the neighborhood of the mask boundary, guiding the mask to avoid regions with high registration errors, thereby effectively suppressing visual artifacts caused by registration residuals.

#### Composite loss.

The final composite loss function used in this paper is formulated as a weighted sum of these three above-mentioned losses, given by:


$$\begin{array}{c} \begin{array}{c} ℒ={ℒ}_{extent}+{ℒ}_{smooth}+{ℒ}_{quality}. \end{array} \end{array}$$ (6)

Note that the three proposed losses have the same weight, and using different weights is out of scope of this paper.

### Accelerated inference via scale decoupling

High-resolution UAV imagery captures rich structural details, yet its massive data volume imposes significant computational and memory demands. To enhance efficiency and reduce resource consumption, existing methods often resort to image downsampling or network simplification. However, these strategies typically sacrifice high-frequency information, degrade model performance, and ultimately compromise the visual fidelity of the stitched results. This inherent trade-off between accuracy and efficiency undermines practical deployment, severely limiting the scalability and real-world utility of high-resolution remote sensing imagery.

The method introduced in Fig 2 employs a neural network to learn multi-level differences between a pair of input images and generates a binary stitching mask accordingly. The final stitched result is obtained by element-wise multiplication of the mask with the corresponding input image and of its complement with the other, followed by summation, avoiding direct fusion of RGB pixels. This design not only improves stitching quality but also paves the way for substantial computational savings. Specifically, the binary nature of the mask ensures that its geometric structures, such as seam boundaries and region partitions, remain stable under downscaling and subsequent upsampling. In stark contrast, RGB images subjected to the same resolution scaling suffer severe high-frequency degradation and irreversible detail loss. Leveraging this scale robustness, we generate the binary mask at a reduced resolution and recover it to full resolution via simple upsampling followed by thresholding. The resulting high-resolution mask is then applied to the original full-resolution input images through element-wise multiplication and summation to produce the final stitched result, achieving substantial computational savings without compromising stitching accuracy.

To quantitatively validate this observation, we employ Shannon’s information entropy as a measure of information stability across scales. For a discrete source *X*, its entropy is defined as:


$$\begin{array}{c} H(X)=-\sum_{i\in X}p_{i}\log_{2}p_{i}\;, \end{array}$$ (7)

where $p_{i}$ denotes the probability of pixel value *i* occurring, representing the color distribution of the image.

For a binary mask *X*={0, 1}, the maximum entropy is 1 bit/pixel, achieved when $p_{0}=p_{1}=0.5$. During upsampling, although bilinear interpolation produces intermediate continuous values, subsequent thresholding restores binarity and preserves the original pixel distribution. As a result, the entropy change $\Delta H_{\text{mask}}=|H_{\text{ori}}-H_{\text{up}}|$ remains approximately zero.

In contrast, an RGB image has three 8-bit channels, yielding a theoretical maximum entropy of 24 bits/pixel. Due to interpolation under the local smoothness assumption, upsampling introduces additional color variations in RGB space. With *N* denoting the upsampling factor, the total number of generated pixels grows as *N*^2^, and experimental results show that the resulting entropy increase $\Delta H_{\text{RGB}}$ approximately follows a quadratic trend with respect to *N*. This marked difference indicates that binary masks have a significant advantage in information stability during upsampling at the same magnification.

Based on this insight, this paper proposes Accelerated Inference via Scale Decoupling, which decouples expensive nonlinear mask prediction from efficient linear blending by allocating them to different resolution spaces. Specifically, the pretrained unsupervised mask network operates on downsampled inputs to generate a coarse binary mask. Thanks to the intrinsic scale robustness of binary structures, this low-resolution mask preserves the essential geometric structure required for accurate stitching, as shown in our entropy-based analysis. The mask is then upsampled and thresholded to produce a full-resolution binary stitching mask, which is used to perform linear blending of the original high-resolution images. This design preserves fine-grained details while avoiding costly nonlinear computation at full resolution, all without requiring network retraining or architectural changes.

In summary, AISD leverages the scale robustness of binary masks to shift nonlinear inference to low resolution space while preserving full resolution output quality through simple linear fusion.

## Results

### Experimental data

The experimental data comprise training and testing datasets. For training, we utilize 10,440 image pairs of 512×512 pixels from the UDIS-D dataset [42]. This training set encompasses image pairs with diverse overlap ratios and parallax magnitudes, covering a broad spectrum of scenes (e.g., indoor, outdoor, nighttime, low-light, snowy, and scaled environments). Such diversity ensures the model learns scale-invariant and scene-robust features. The absence of aerial images in the training set enables cross-domain evaluation of generalization performance. For testing, we employ two public UAV datasets and two self-collected UAV datasets. All test images are characterized by large spatial dimensions and significant parallax, posing substantial challenges for image stitching. Each dataset also presents unique difficulties that further probe the robustness of our method. Detailed characteristics of the four datasets are summarized in Table 1.

**Table 1:** Image Size and characteristics of the test datasets.

Dataset	Image Size	Characteristics
Urban [50]	6000×4000	Salient structural features, large parallax, partial occlusions.
Highway [50]	7952×5304	Prominent structures and moving vehicles
Town1	6000×4000	Densely distributed buildings, roads, weakly textured woodland, and farmland.
Town2	8192×5460	Densely distributed buildings and roads, partial occlusions.

### Experimental settings and training details

The proposed method is implemented in PyTorch 1.10 with Python 3.9. The model contains 41.79 M trainable parameters and is trained using the Adam optimizer with an initial learning rate of $1\times 10^{-5}$ and a batch size of 1. Training is conducted on a single NVIDIA GeForce RTX 3090 GPU (24 GB memory) for 200 epochs, taking approximately two weeks. This duration was chosen because validation performance continues to improve beyond 150 epochs and stabilizes around 180 epochs, yielding optimal results. For testing, we use an Intel i5-10400F CPU (2.90 GHz) with 40 GB RAM, without GPU acceleration.

### Baseline methods

To comprehensively evaluate the proposed method, we compare it against several representative image stitching approaches, including both classical methods and recent deep learning-based techniques. All methods are implemented using their publicly available code with recommended settings. The details of these baselines are summarized as follows:

- ORB [51] is a fast-stitching method that uses ORB features [52] for description and global alignment for image registration. In this experiment, the method is implemented using GitHub code [51].
- APAP [16] and SPW [19] are classic image stitching methods that use grid-based warping for local alignment.
- ELA [17] employs thin-plate splines to model elastic deformations for image stitching.
- LPC [20] is an image stitching method designed to preserve image content integrity, incorporating graph-cut-based optimal seam finding.
- UDIS++ [43] is an unsupervised deep image stitching framework that comprises both an image alignment network and an image synthesis network. It contains 8.23 M parameters.

### Visual comparison with state-of-the-art image stitching methods

In this experiment, we first visually compare our method with the aforementioned baselines. This comparison aims to evaluate the visual quality of our proposed method against both classical and state-of-the-art image stitching techniques. To comprehensively assess performance, we select a diverse set of challenging scenarios from the test datasets, focusing on three representative cases that pose significant difficulties for seamless stitching:

(1) Scenes with large parallax: Common in UAV-captured imagery, substantial viewpoint shifts between adjacent frames lead to pronounced parallax, which severely complicates geometric alignment and often results in misregistration or ghosting artifacts.
(2) Scenes with moving objects: Dynamic elements such as vehicles or boats change position across multi-temporal captures. These inter-frame displacements introduce inconsistencies that conventional methods struggle to reconcile, frequently yielding duplicated structures or blurred transitions.
(3) Scenes with large rotation and dense urban structures: During typical S-shaped UAV flight paths, particularly during heading reversals or cross-track imaging, adjacent views exhibit significant rotational differences. Combined with the complex 3D geometry of buildings and vegetation, this leads to severe occlusions and perspective distortions, presenting a formidable challenge for accurate stitching.

To facilitate detailed visual assessment, this paper present qualitative results for these scenarios alongside the corresponding input images and zoomed-in patches. In all figures in the following parts, each column corresponds to a specific stitching method, labeled at the top. The second row shows a thumbnail of the full stitched panorama, while subsequent rows display localized regions that emphasize common artifacts—including blurring, structural misalignment, and geometric distortion. For reference, the rightmost column provides ground-truth patches cropped from the reference image. Red arrows indicate blurring, and yellow arrows mark structural misalignments.

The results are organized by scenario: First, evaluate large-parallax cases; then, address scenes with moving objects; finally, focus on complex urban environments featuring large rotations and dense structures.

Visual inspection in the following parts demonstrates that, across all challenging scenarios evaluated in this study, the proposed method consistently achieves high visual fidelity and effectively suppresses blurring, misalignment, and geometric distortions.

#### Large-parallax scenario.

**Fig 7:**
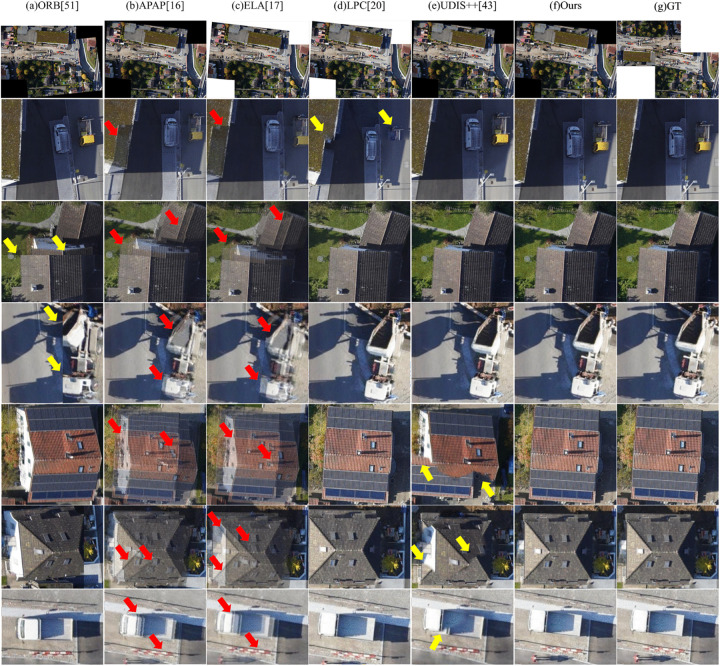
Qualitative comparison of image stitching methods on large-parallax scenario – Example 1.

**Fig 8:**
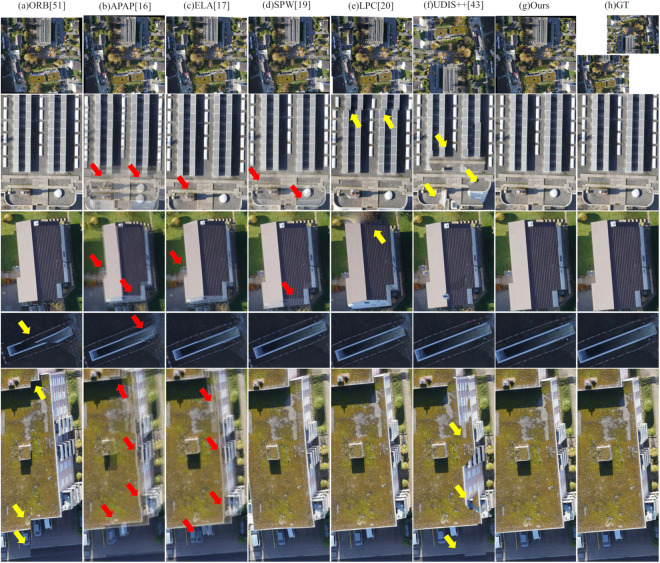
Qualitative comparison of image stitching methods on large-parallax scenario – Example 2.

Figs 7 and 8 present visual comparisons of different image stitching methods on scenes with large parallax. One can observe that the performance of these methods varies significantly under such challenging conditions:

First, the ORB method exhibits notable geometric misalignment at the image seams. For instance, the roof of a house is fractured in the third row of Fig 7, a construction vehicle is partially truncated in the fourth row, and building edges are clearly misaligned in the fourth and fifth rows of Fig 8.

Second, both APAP and ELA results contain multiple prominent artifacts, particularly in handling building lines. As shown in the third, fifth, and sixth rows of Fig 7, and the third to fifth rows of Fig 8, the building lines suffer from ghosting or fractures. Additionally, the edges of a truck in the seventh row of Fig 7 display obvious artifacts and blurring.

Third, LPC achieves relatively higher stitching quality, yet local structural misalignments still exist. Examples include fractured building edges and a partially missing construction vehicle in the second row of Fig 7, as well as misaligned solar panels in the second row of Fig 8 and discontinuous building edges in the third row.

Fourth, UDIS++ demonstrates significant structural discontinuity. Examples include large-scale, noticeable misalignments in building regions shown in the fifth and sixth rows of Fig 7, and the second and fifth rows of Fig 8. The truck edge in the seventh row of Fig 7 is also misaligned.

In contrast, our method (Ours) effectively reduces artifacts and geometric misalignments while maintaining global consistency, producing clear and complete stitching results. Both the continuity of building lines and the edge structures of objects such as trucks in our results closely align with the ground truth (GT), demonstrating superior robustness and higher visual quality.

#### Moving objects scenario.

**Fig 9:**
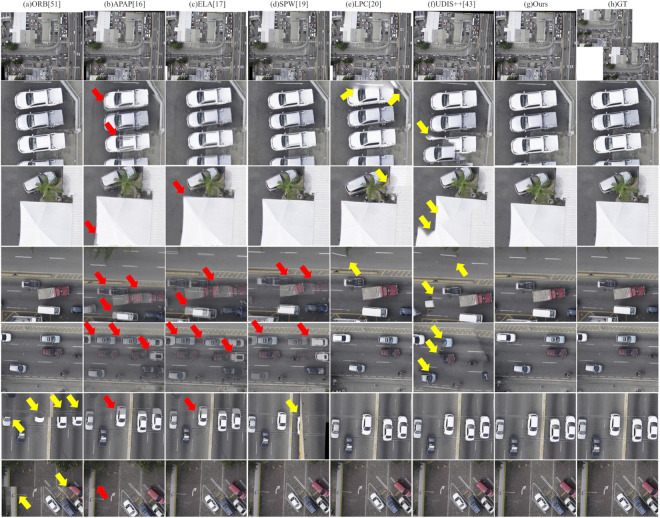
Qualitative comparison of image stitching methods on moving objects scenario – Example 1.

**Fig 10:**
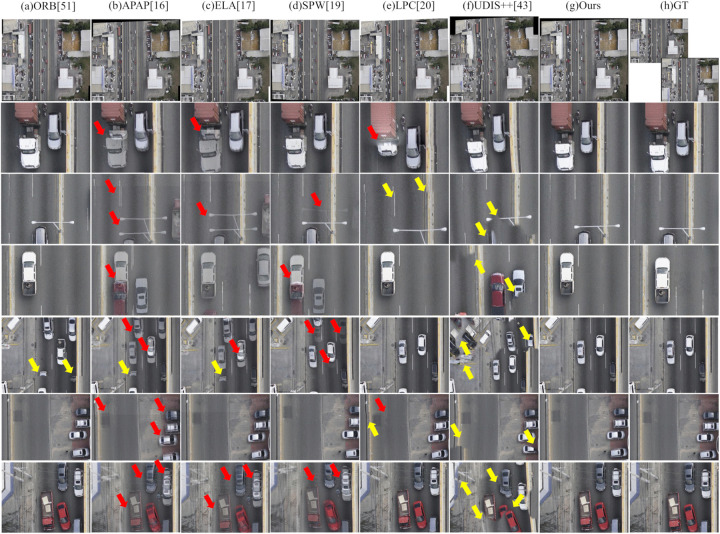
Qualitative comparison of image stitching methods on moving objects scenario – Example 2.

Figs 9 and 10 present visual comparisons of different image stitching methods on scenes with dense moving objects. One can observe that the performance of these methods varies significantly under such dynamic conditions:

First, the ORB method exhibits notable geometric misalignment at the image seams. This is because its pipeline of global alignment followed by direct fusion increases the risk of truncating moving objects in dynamic scenes. As indicated by the yellow arrows in Figs 9 and 10, moving cars are cleanly truncated and road markings are clearly misaligned.

Second, similar to large-parallax scenes, significant artifacts also appear in the stitching results of APAP, ELA, and SPW. Not only do stationary objects such as the car and buildings in Fig 9 exhibit obvious edge artifacts, but dynamic objects introduce even more severe distortions. For instance, in Figs 9 and 10, moving cars commonly suffer from severe ghosting or tearing. Particularly in these figures, the dense and inconsistent motion of vehicles leads to multiple overlaps in the output, rendering the stitching results visually chaotic and severely affecting the overall viewing experience.

Third, LPC demonstrates high overall stability and effectiveness under the evaluated conditions, producing virtually no visible artifacts or severe misalignments. Nevertheless, minor structural misalignments can still be observed at several locations indicated by yellow arrows in Figs 9 and 10, including along the edges of vehicles, road markings, and buildings.

Fourth, UDIS++ also produces severe geometric misalignments in its stitched results. In Fig 9, both stationary vehicles and buildings show noticeable structural distortions. Moreover, in Figs 9 and 10, multiple moving vehicles are only partially preserved, with some parts of the bodies truncated or missing, resulting in fragmented appearances. This significantly compromises the visual realism and completeness of the stitched image.

In contrast, the proposed method maintains relatively good stitching quality even in scenes with dense dynamic objects. The resulting images preserve sharp edges, geometric consistency, and natural visual transitions. As shown in Figs 9 and 10, both static and moving objects retain well-defined contours, free from noticeable artifacts, ghosting, or geometric misalignments.

#### Large-rotation and dense buildings scenario.

**Fig 11:**
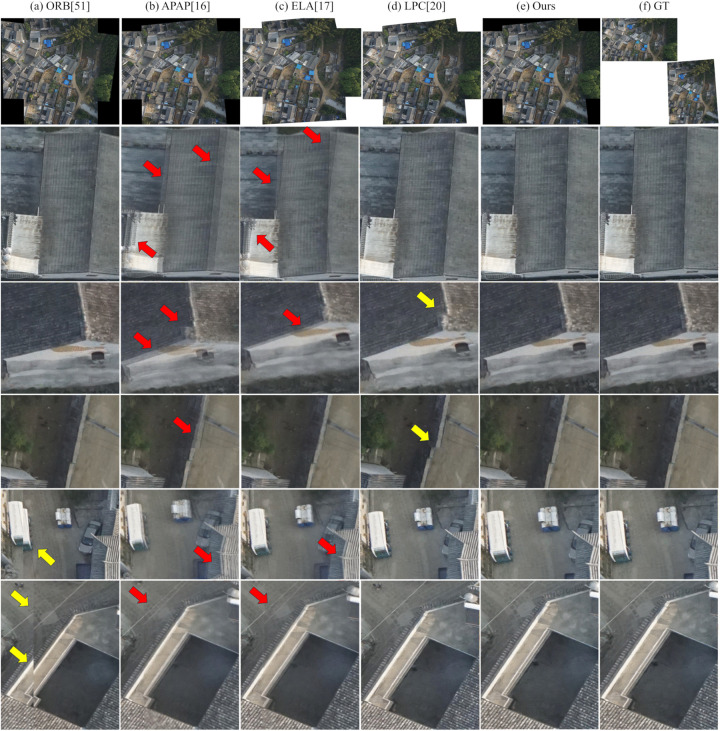
Qualitative comparison of image stitching methods on large rotation and dense buildings scenario – Example 1.

**Fig 12:**
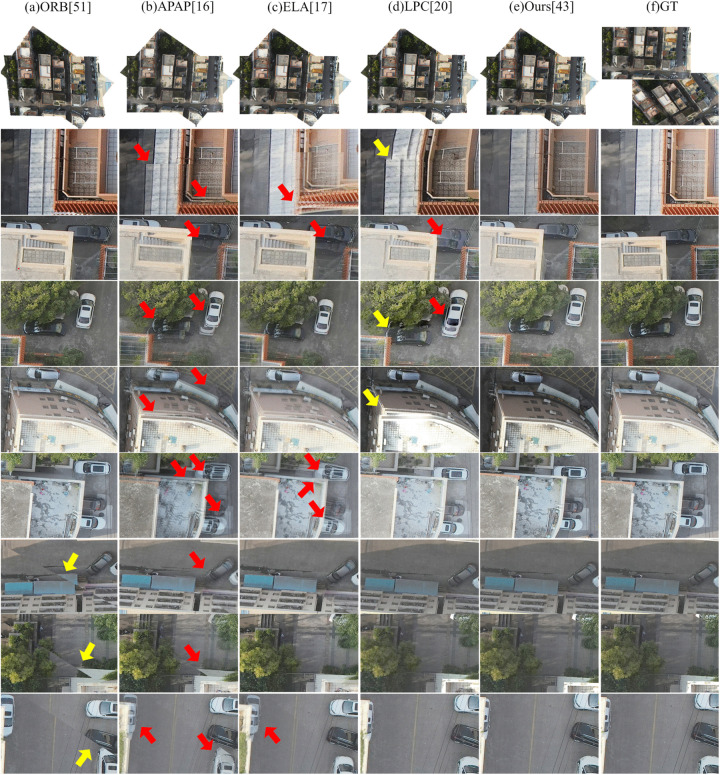
Qualitative comparison of image stitching methods on large rotation and dense buildings scenario – Example 2.

Figs 11 and 12 present visual comparisons of different image stitching methods on scenes with large rotation angles and dense buildings. Note that the results of UDIS++ and SPW are not presented because of their poor performance and a high failure rate under large rotation conditions.

Under such conditions, the performance of these methods varies significantly:

First, the ORB method produces a visible stitching seam at the junction of the two images, resulting in an abrupt transition that severely disrupts visual continuity. Similar issues (e.g., truncated objects and misaligned markings) are also observed in the previous two scenes, and they become even more pronounced in scenarios with large rotation angles and dense urban structures.

Second, both APAP and ELA can achieve image alignment to some extent, yet they still exhibit noticeable artifacts. Specifically, as indicated by the red arrows in Figs 11 and 12, significant blurring artifacts can be observed along multiple building edges (in both figures) and along several vehicles in Figs 12, compromising structural sharpness and local detail preservation.

Third, LPC demonstrates better structure preservation compared to APAP and ELA. However, it still suffers from slight misalignments at building and road boundaries, indicating room for improvement in handling complex urban geometries under large rotations.

Finally, the proposed method outperforms all competing methods in preserving structural integrity and edge sharpness. Straight-line structures remain continuous and natural, with sharp and clear edges, significantly improving both the overall visual quality and geometric fidelity.

### Quantitative evaluation

Experimental results in Tables 2 and 3 demonstrate that the proposed method consistently outperforms all competing methods (ORB [51], APAP [16], ELA [17], SPW [19], LPC [20], and UDIS++ [43]) in terms of both PSNR and SSIM across all four datasets.

**Table 2:** Quantitative comparison of PSNR (dB) with baselines.

Dataset	ORB [51]	APAP [16]	ELA [17]	SPW [19]	LPC [20]	UDIS++ [43]	Ours
Urban	13.62	21.68	22.48	17.28	18.77	18.39	**23.20**
Highway	15.52	22.52	26.10	24.56	25.93	20.02	**27.93**
Town1	16.65	17.52	15.01	15.98	17.01	14.73	**18.95**
Town2	11.32	18.32	17.43	17.90	23.54	20.43	**24.52**

**Table 3:** As Table 2 but for SSIM.

Dataset	ORB [51]	APAP [16]	ELA [17]	SPW [19]	LPC [20]	UDIS++ [43]	Ours
Urban	0.23	0.63	0.69	0.34	0.37	0.45	**0.73**
Highway	0.37	0.56	0.71	0.61	0.73	0.59	**0.79**
Town1	0.27	0.35	0.40	0.35	0.45	0.26	**0.51**
Town2	0.20	0.38	0.41	0.45	0.63	0.50	**0.70**

Compared to the second-best method in each dataset (ELA on Urban and Highway, APAP on Town1, and LPC on Town2), our method achieves average PSNR gains of 1.24 dB and average SSIM gains of 0.058. Specifically, the PSNR improvements over the respective second-best results are 0.72 dB(Urban), 1.83 dB(Highway), 1.43 dB(Town1) and 0.98 dB(Town2). And the SSIM improvements are 0.04, 0.06, 0.06, and 0.07, respectively.

Overerall, these results confirm the superiority of the proposed method in UAV image stitching. It not only significantly enhances the quality of the stitched images but also demonstrates strong robustness and efficiency across various complex and challenging conditions.

### Stitching time comparison

Table 4 reports the runtime (in seconds) of our method against four baselines (ORB [51], APAP [16], ELA [17] and UDIS++ [43]) on four datasets with varying image resolutions. The methods SPW [19] and LPC [20] are excluded from this comparison, as their single-pair stitching time consistently exceeds 60 minutes, rendering comparison under large-scale, high-resolution conditions impractical.

**Table 4:** Stitching time comparison of our method and baselines (in seconds).

Dataset	Image Size	ORB [51]	APAP [16]	ELA [17]	UDIS++ [43]	Ours
Urban	6000×4000	7.1	720.0	505.2	108.1	56.7
Highway	3976×2652	5.9	261.3	210.0	191.5	25.4
Town1	3000×2000	4.5	108.3	72.4	42.8	16.9
Town2	8192×5460	10.7	1626.2	1415.2	N/A	169.9

On the three datasets where all methods produced valid results (Urban, Highway, and Town1), the proposed method achieves average time reductions of 88.9%, 84.5%, and 64.9% compared to APAP, ELA, and UDIS++, respectively. On the highest-resolution Town2 dataset, the proposed method successfully processes the image pairs in 169.9 seconds, reducing runtime by 89.6% and 88.0% compared to APAP and ELA, respectively, while UDIS++ fails to complete the stitching task.

Although ORB yields much lower processing times due to its straightforward pipeline of global alignment followed by direct fusion without seam handling, its poor stitching quality with visible seams limits its practical utility in high-fidelity applications.

The efficiency advantage of the proposed method is primarily attributed to the synergistic interaction between its unique AISD strategy and the unsupervised mask generation mechanism. By decoupling the mask generation process from image resolution, computationally intensive operations are effectively confined to the low-resolution domain, thereby breaking the strong dependence of computational complexity on resolution that characterizes conventional approaches. This strategy preserves high stitching accuracy while reducing the processing time per image pair by an order of magnitude. Together, these results validate the engineering applicability of the proposed method, particularly in large-scale, high-resolution UAV image stitching scenarios where both efficiency and robustness are critical.

### Computational efficiency analysis with scale decoupling

To evaluate the effectiveness of the proposed Accelerated Inference via Scale Decoupling strategy, an ablation study is conducted focusing on this strategy. Table 5 presents the average stitching time of the proposed method under different image downsampling ratios. One can see that, as the image resolution decreases, the stitching time drops significantly, leading to improved computational efficiency. Tables 6 and 7 present the PSNR and SSIM values of stitching results under different downsampling factors, respectively, demonstrating that despite significant variations in image resolution, the objective quality metrics remain relatively stable.

**Table 5:** Stitching time (s) for different downsampling ratios.

Dataset	Image Size	10%	30%	50%	70%
Urban	6000×4000	56.7	88.3	130.1	N/A
Highway	3976×2652	25.4	43.1	51.3	81.2
Town1	3000×2000	16.9	25.8	34.2	55.1
Town2	8192×5460	169.9	278.1	340.7	N/A

**Table 6:** PSNR (dB) of stitched images at different downsampling scales.

Dataset	10%	30%	50%	70%
Urban	23.20	23.19	23.22	N/A
Highway	27.93	27.91	27.90	27.91
Town1	18.95	19.02	19.01	19.06
Town2	24.52	24.40	24.48	N/A

**Table 7:** As Table 6 but for SSIM.

Dataset	10%	30%	50%	70%
Urban	0.73	0.72	0.71	N/A
Highway	0.84	0.83	0.82	0.82
Town1	0.51	0.50	0.48	0.49
Town2	0.70	0.69	0.71	N/A

## Discussion

### Discussion of results

The proposed unsupervised deep learning framework effectively addresses the challenges of large parallax and complex dynamic backgrounds in UAV image stitching. By reformulating seam removal as an end-to-end pixel-wise prediction task, our method demonstrates the ability to produce visually natural and seamless stitchings in highly complex scenes, overcoming the inherent constraints of traditional optimization-based approahes.

This study introduces three methodological contributions that together constitute the key advantages of our approach. First, the reformulation of image stitching as a dense pixel-wise prediction task fundamentally expands the solution space compared to traditional one-dimensional seam search methods. By treating each pixel in the overlapping region as an independent classification problem, our framework achieves greater flexibility in handling depth discontinuities and dynamic objects, effectively avoiding the topological rigidity that limits conventional optimal seam algorithms. Second, the unsupervised learning paradigm enabled by our Siamese-Residual Mask Network (SRMN) with three physics-informed loss functions eliminates the reliance on hand-crafted features and energy functions that characterize traditional methods, while also circumventing the annotation dependency inherent in supervised deep learning approaches. This allows the model to learn directly from data and adapt to diverse scene complexities without manual intervention. Third, the scale-decoupled inference strategy preserves fine-grained structural details while avoiding the computational burden of full-resolution nonlinear processing. Based on the observation that binary masks maintain geometric integrity under resolution scaling, this strategy allocates nonlinear mask prediction and linear blending to different resolution spaces, achieving efficient inference without requiring network retraining or architectural modifications.

### Limitations

While the proposed SRMN demonstrates promising performance in UAV image stitching, it is important to acknowledge that it still has its limitations.

First, the SRMN has approximately 41.79 M parameters, which poses challenges for real-time UAV image stitching on resource-constrained platforms. Future work could focus on designing more lightweight network architectures [53] to reduce computational costs while maintaining stitching quality.

Second, while SRMN performs well under standard conditions, its reliance on manually predefined loss weights limits its generalizability across highly diverse and extreme scenarios, such as severe low-light environments. In such cases, static coefficients are probably insufficient to balance the competing loss terms. Future research could focus on studying adaptive optimization strategies, including reinforcement learning [54] or game theory [55,56], to improve performance in challenging operational contexts.

## Conclusion

This paper proposes an unsupervised and mask-based image stitching method for UAV remote sensing, which reframes stitching as a 2D dense prediction problem and introduces two key components: Siamese-Residual Mask Network (SRMN) and Accelerated Inference via Scale Decoupling (AISD). Comprehensive experiments on four UAV datasets demonstrate the effectiveness of the proposed method.

In terms of stitching quality, compared with all baselines (ORB, APAP, ELA, SPW, LPC, UDIS++), the proposed method improves average PSNR by 10.97%–65.64% and average SSIM by 23.53%–155.14%. Compared with state-of-the-art methods only (APAP, ELA, SPW, LPC, UDIS++), the average improvements are 10.97%–28.58% for PSNR and 23.53%–56.00% for SSIM. Although ORB is faster, its stitching quality is substantially lower, limiting its practical utility. In terms of computational efficiency, the method reduces average stitching time by 64.9%–88.9% while robustly handling ultra-high-resolution images where other methods fail.

These results confirm that the proposed method effectively balances stitching quality and efficiency, making it well-suited for large-scale, real-world UAV image stitching. Future work includes developing lightweight network architectures and adaptive optimization strategies to further improve generalization.
